# Repetitive magnetic stimulation induces plasticity of inhibitory synapses

**DOI:** 10.1038/ncomms10020

**Published:** 2016-01-08

**Authors:** Maximilian Lenz, Christos Galanis, Florian Müller-Dahlhaus, Alexander Opitz, Corette J. Wierenga, Gábor Szabó, Ulf Ziemann, Thomas Deller, Klaus Funke, Andreas Vlachos

**Affiliations:** 1 Institute of Clinical Neuroanatomy, Neuroscience Center, Goethe-University, Frankfurt/Main 60590, Germany; 2 Department of Neurology and Stroke and Hertie Institute for Clinical Brain Research, Eberhard-Karls-University, Tübingen 72076, Germany; 3 Center for Biomedical Imaging and Neuromodulation, Nathan Kline Institute for Psychiatric Research, Orangeburg, New York 10962, USA; 4 Center for the Developing Brain, Child Mind Institute, New York, New York 10022, USA; 5 Division of Cell Biology, Faculty of Science, Utrecht University, Utrecht 3584 CH, The Netherlands; 6 Laboratory of Molecular Biology and Genetics, Institute of Experimental Medicine, Budapest H1083, Hungary; 7 Department of Neurophysiology, Medical Faculty, Ruhr-University, Bochum 44780, Germany

## Abstract

Repetitive transcranial magnetic stimulation (rTMS) is used as a therapeutic tool in neurology and psychiatry. While repetitive magnetic stimulation (rMS) has been shown to induce plasticity of excitatory synapses, it is unclear whether rMS can also modify structural and functional properties of inhibitory inputs. Here we employed 10-Hz rMS of entorhinohippocampal slice cultures to study plasticity of inhibitory neurotransmission on CA1 pyramidal neurons. Our experiments reveal a rMS-induced reduction in GABAergic synaptic strength (2–4 h after stimulation), which is Ca^2+^-dependent and accompanied by the remodelling of postsynaptic gephyrin scaffolds. Furthermore, we present evidence that 10-Hz rMS predominantly acts on dendritic, but not somatic inhibition. Consistent with this finding, a reduction in clustered gephyrin is detected in CA1 stratum radiatum of rTMS-treated anaesthetized mice. These results disclose that rTMS induces coordinated Ca^2+^-dependent structural and functional changes of specific inhibitory postsynapses on principal neurons.

## Abstract (web summary)

Repetitive transcranial magnetic stimulation (rTMS) is widely used as a therapeutic tool yet its effect on inhibitory networks in the brain has not been studied. Here, the authors demonstrate that 10Hz rTMS specifically reduces dendritic inhibition onto pyramidal neurons accompanied by remodeling of postsynaptic gephyrin clusters.

## Graphical abstract

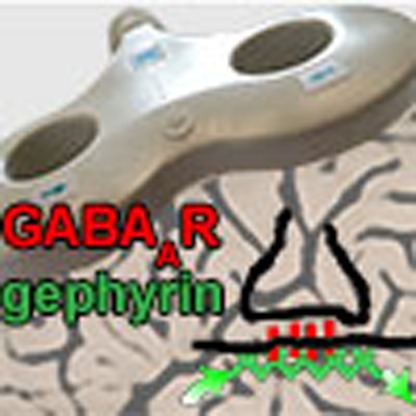

The therapeutic potential of repetitive transcranial magnetic stimulation (rTMS) has been extensively studied in the context of brain diseases, such as addiction, depression, Parkinson's disease, schizophrenia and stroke^1^. Yet, the cellular and molecular mechanisms underlying rTMS-based therapies remain not well understood^2^,^3^. Thus, a better understanding of rTMS-induced neural plasticity is needed to optimize treatment protocols and to develop new diagnostic and therapeutic strategies using rTMS. Studies in suitable animal models or in appropriate *in vitro* preparations provide experimental approaches in this context, since electrophysiological, (live-cell) imaging and molecular biology techniques can be combined with genetic and pharmacologic methods to unravel how repetitive magnetic stimulation (rMS) induces neural plasticity^4^.

In our previous work we were able to demonstrate that rMS of mouse entorhinohippocampal slice cultures leads to long-lasting structural and functional changes of excitatory postsynapses^5^,^6^. Using a 10-Hz rMS protocol we observed a robust strengthening of excitatory inputs and the enlargement of dendritic spines on CA1 pyramidal neurons (2–4 h post-magnetic stimulation, pms)^5^. Since these changes were mediated by N-methyl-D-aspartate receptors (NMDARs), the results of this earlier study indicated that rMS is capable of inducing long-term potentiation (LTP) of α-amino-3-hydroxy-5-methyl-4-isoxazolepropionic acid receptor-mediated synaptic transmission^5^. Furthermore, we recently showed that the effects of rMS on excitatory synapses occur predominantly on proximal dendrites of cultured CA1 pyramidal neurons^6^. Hence, rMS may induce plasticity of specific excitatory synapses of a neuron.

Considering recent experimental evidence, which indicates a crucial role for inhibitory synaptic transmission in cortical network function^7^,^8^,^9^ and the suggestion that alterations in excitation and inhibition (E/I) balance underlie states of behavioural and cognitive dysfunction in brain diseases^10^,^11^,^12^,^13^, we here sought to determine the effects of the same 10-Hz rMS protocol^5^,^6^ on structural and functional properties of inhibitory synapses. Gephyrin, the major postsynaptic scaffolding protein to which ionotropic γ-aminobutyric acid receptors (GABA_A_R) anchor^14^,^15^,^16^, was used to test for rMS-induced structural changes of inhibitory postsynaptic sites on CA1 pyramidal neurons^17^. Our results disclose a reduction in inhibitory synaptic strength, which is accompanied by the destabilization of gephyrin clusters following rMS *in vitro* (2–4 h after stimulation). These structural and functional changes require the activation of voltage-gated sodium channels (VGSCs), L-type voltage-gated calcium channels (L-VGCCs) and NMDARs, and are not observed when calcineurin protein phosphatases are pharmacologically blocked. While the Ca^2+^-dependent cysteine protease (calpain) inhibitor MDL-28170 (50 μM)^18^ has no effect in our experiments, the results of the present study corroborate the previously proposed gephyrin-mediated Ca^2+^/calcineurin-dependent tuning of inhibitory synapses, which accompanies LTP of excitatory synapses^19^. Notably, dendritic inhibition appears to be mainly affected in our experimental setting, suggesting that 10-Hz rMS may not act equally on all inhibitory synapses of a neuron. Consistent with this finding, a reduction in gephyrin cluster sizes and numbers is observed in the dendritic layer, that is, CA1 stratum radiatum of 10-Hz rTMS-treated anaesthetized mice. Together with our previous work^5^,^6^, we propose that rMS acts on network excitability and connectivity through the induction of coordinated, Ca^2+^-mediated changes of specific subsets of excitatory and inhibitory synapses on principal neurons.

## Results

### rMS of organotypic slice cultures

**Figure 1:**
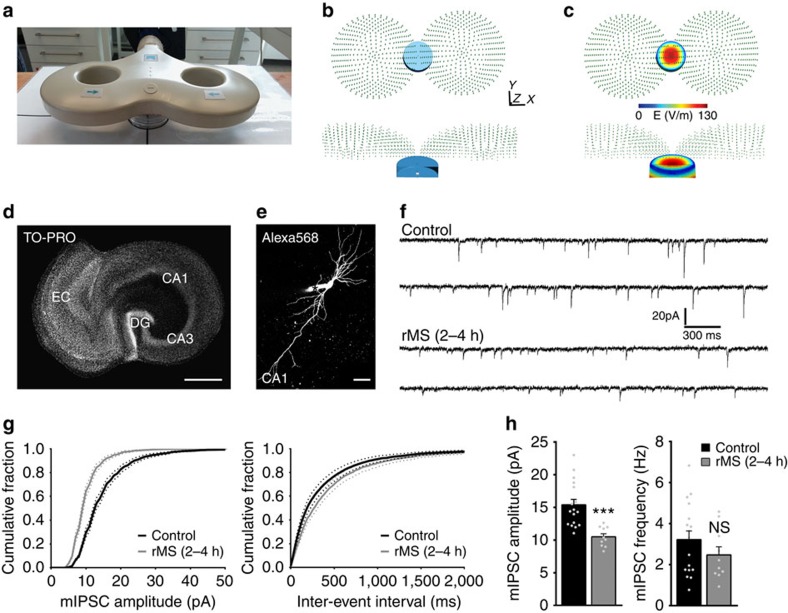
rMS of mouse entorhinohippocampal slice cultures induces a reduction in inhibitory synaptic transmission. (**a**) A photograph illustrating the experimental setting. Slice cultures (Petri dish below the coil) are stimulated with a standard 70-mm outer wing diameter figure-of-eight coil. Distance from coil, position and orientation of cultures within the magnetic field are kept constant in all experiments^5^,^6^. (**b**,**c**) The electric field induced by rMS *in vitro* was described based on a magnetic dipole model of the 70-mm figure-of-eight coil^62^. A finite element method model was created with the dimensions of the bath (blue) and tissue (grey) adapted to the *in vitro* setting. The coil was positioned 1 cm above the tissue (**b**). The electric field induced in our experimental setting was estimated to be 27.3±3.3 V m^−1^ in the tissue (**c**), which is within the lower range of clinical TMS motor-evoked potential threshold amplitudes of 30–130 V m^−1^ (ref. 68). (**d**) Entorhinohippocampal slice culture stained with TO-PRO nuclear stain. All cultures were oriented in the same direction. Four to six cultures were stimulated at the same time (EC, entorhinal cortex; DG, dentate gyrus). Scale bar, 500 μm. (**e**) Patched CA1 pyramidal neuron filled with Alexa568. Asterisk indicates the Alexa568 (10 μM) containing tip of the patch pipette. Scale bar, 50 μm. (**f**) Sample traces of mIPSCs recorded from CA1 pyramidal neurons in non-stimulated control cultures and cultures 2–4 h after rMS. (**g**,**h**) Cumulative distributions of mIPSC amplitudes and interevent intervals (**g**). A significant reduction in the mean mIPSC amplitude but not the mean mIPSC frequency is observed 2–4 h after stimulation (h; control, *n*=17 neurons; rMS, *n*=10 neurons from five cultures each; Mann–Whitney test). Individual data points are indicated by gray dots. Values represent mean±s.e.m. (***P<0.001; ns, not significant differences).

Slice cultures (⩾18 days *in vitro*) containing the entorhinal cortex and the hippocampus were stimulated using a standard 70-mm figure-of-eight coil (Fig. 1a)^5^,^6^. The electric field strength induced in the slice culture preparations was described using computational modelling (27.3±3.3 V m^−1^; Fig. 1b,c). Stimulated and non-stimulated (but otherwise equally treated) cultures were returned to the incubator and experimentally assessed at 2–4 h pms, which is the time period during which structural and functional changes of excitatory synapses are most prominently observed in our experimental setting^5^.

### rMS induces changes in inhibitory synaptic transmission

**Figure 2:**
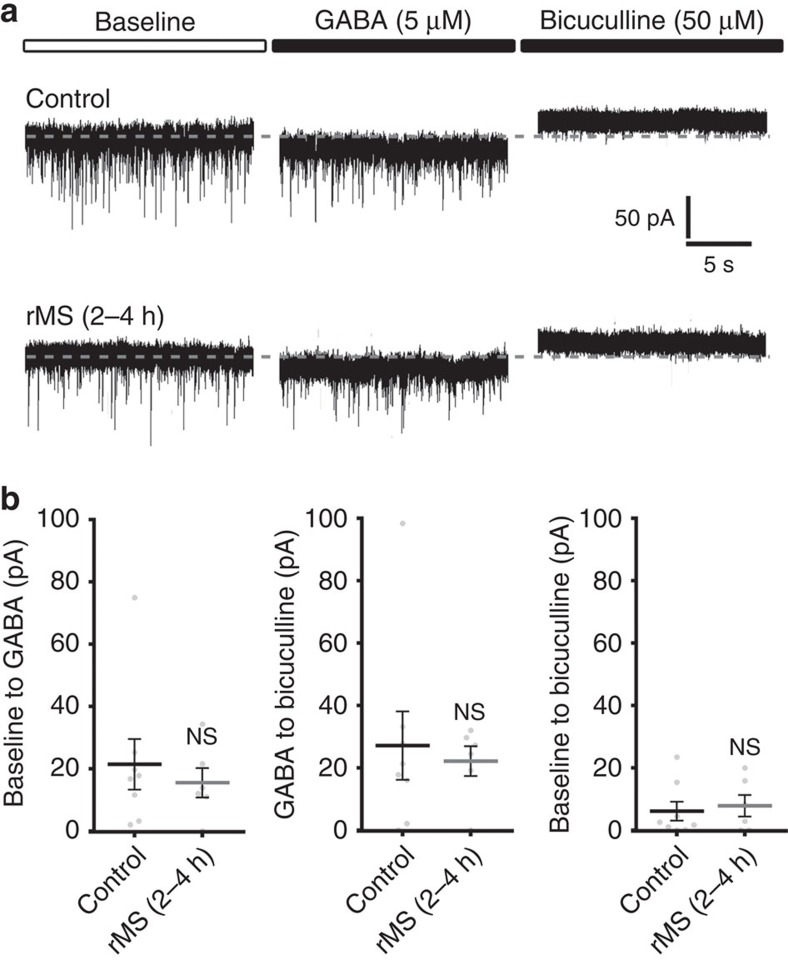
Tonic GABA_A_R conductance is not affected after rMS. (**a**) Sample traces depicting GABA- (5 μM) and bicuculline-methiodide (50 μM)-induced shift in tonic GABA_A_R-mediated currents recorded from CA1 pyramidal neurons in non-stimulated control and stimulated slice cultures (2–4 h after rMS). Baseline currents were not significantly different between the two groups (control: 191.2±12.1 pA; rMS: 187.5±13.6 pA; *P*=0.57; control, *n*=8 neurons; rMS, *n*=6 neurons; one cell per culture; Mann–Whitney test). (**b**) The amplitude of GABA- or bicuculline-methiodide-induced shifts in tonic GABA_A_R currents of CA1 pyramidal neurons is not significantly different between stimulated and non-stimulated slice cultures (control, *n*=8 neurons; rMS, *n*=6 neurons; one cell per culture; Mann–Whitney test). Individual data points are indicated by grey dots. Values represent mean±s.e.m. (ns, not significant differences).

To determine rMS-induced changes in inhibitory synaptic strength we patched individual CA1 pyramidal neurons and recorded miniature inhibitory postsynaptic currents (mIPSCs) in whole-cell voltage-clamp mode from non-stimulated control and stimulated cultures (Fig. 1d–f). Recordings were performed at a holding potential of −80 mV in the presence of tetrodotoxin (TTX; 0.5 μM), which inhibits VGSCs, as well as inhibitors of NMDARs (D(-)-2-amino-5-phosphonovaleric acid, AP5, 10 μM) and α-amino-3-hydroxy-5-methyl-4-isoxazolepropionic acid receptors (6-cyano-7-nitroquinoxaline-2,3-dione, CNQX, 10 μM). Hence, we assessed properties of GABA_A_R-mediated currents evoked by stochastic release of GABA from presynaptic terminals (Fig. 1f), which could be blocked by the GABA_A_R inhibitor SR95531 (10 μM) or bicuculline-methiodide (50 μM; *c.f.*, Fig. 2a). In these experiments a significant reduction in mIPSC amplitudes was observed after rMS, while mIPSC frequencies were not significantly changed (Fig. 1g,h). Since the amplitude of mIPSCs is considered to reflect postsynaptic strength, we conclude that rMS *in vitro* induces a reduction in GABA_A_R-mediated synaptic transmission on CA1 pyramidal neurons 2–4 h pms.

### rMS has no major effect on tonic inhibition

We then tested for the effects of 10-Hz rMS on extrasynaptic GABA_A_R conductance, which is mediated by ambient GABA in the extracellular space (Fig. 2). A different set of cultures was stimulated and individual CA1 pyramidal neurons were patched 2–4 h pms in presence of TTX, AP5 and CNQX. After obtaining stable baseline recording, GABA (5 μM)-containing extracellular solution was washed into the recording chamber, followed by bicuculline-methiodide (50 μM)-containing extracellular solution to block GABA_A_Rs at the end of each experiment (Fig. 2a). No significant difference between the two groups was observed in (1) the baseline (control: −191.2±12.1 pA, *n*=8 neurons from eight cultures; rMS: −187.5±13.6 pA, *n*=6 neurons from six cultures; *P*=0.57; Mann–Whitney test), (2) the GABA-mediated shift or (3) the bicuculline-induced change in tonic GABA_A_R currents (Fig. 2b). Notably, we were able to confirm a significant reduction in mIPSC amplitudes after rMS in the baseline recordings (mIPSC amplitude, control: 34.4±1.6 pA, *n*=8 neurons from eight cultures; rMS: 28.4±1.7 pA, *n*=6 neurons from six cultures; *P*<0.05; Mann–Whitney test; mIPSC frequency, control: 9.3±0.8 Hz, *n*=8; rMS: 7.6±0.5 Hz, *n*=6; *P*=0.14; Mann–Whitney test; internal solution containing higher [Cl^−^] compared with Fig. 1). Consistent with these findings we did not detect a significant difference in GABA_A_Rα5 immunostainings between stimulated and non-stimulated cultures 3 h after rMS (Supplementary Fig. 1; in line with previous work clustered GABA_A_Rα5 staining was observed^20^,^21^). Although these experiments do not fully exclude the possibility that rMS affects tonic inhibition, the negative immunostaining results for GABA_A_Rα5 (c.f. Supplementary Fig. 1) and the mIPSC results (*c.f.*, Fig. 1) prompted us to focus on rMS-induced changes in phasic, that is, synaptic inhibitory neurotransmission.

### rMS reduces size and number of gephyrin clusters

**Figure 3:**
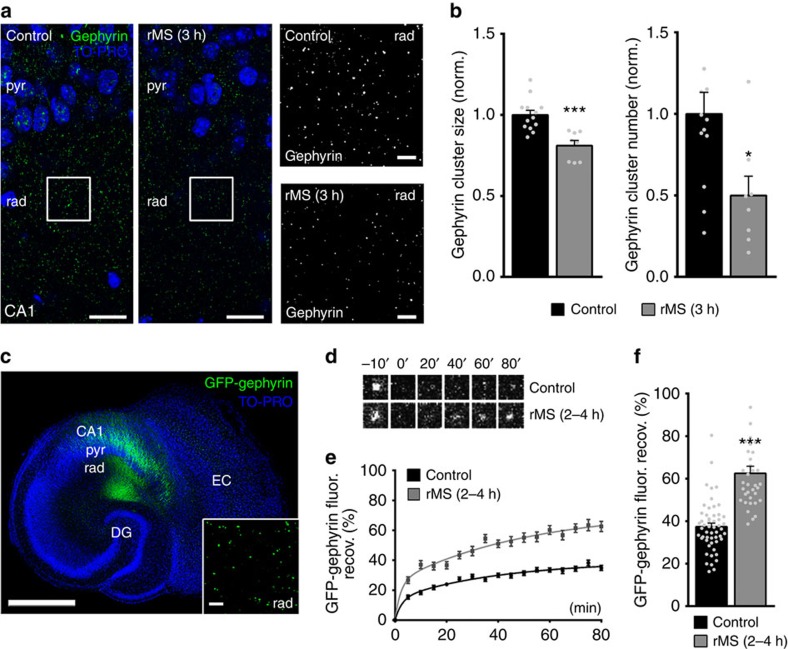
rMS induces changes in gephyrin cluster properties. (**a**) Stimulated and non-stimulated cultures stained for endogenous gephyrin. Cluster sizes and numbers were assessed in the CA1 stratum radiatum (rad). Blue, TO-PRO nuclear stain; pyr, stratum pyramidale. Scale bars, 30 μm (at higher magnification, 4 μm). (**b**) A reduction in the mean gephyrin cluster size and number is observed after rMS. Values normalized to non-stimulated control cultures (control, *n*=13 cultures; rMS, *n*=8 cultures; averaged data from three visual fields per culture; Mann–Whitney test; two data points outside the axis limits). (**c**) Overview of a slice culture prepared from a *Thy1-GFP/gephyrin* mouse. GFP-gephyrin expression (green) is regularly seen in area CA1 in these preparations. FRAP of individual GFP-gephyrin clusters was assessed in the stratum radiatum (rad) of area CA1. Blue, TO-PRO nuclear stain; pyr, stratum pyramidale; DG, dentate gyrus; EC, entorhinal cortex. Scale bar, 500 μm (inset shows GFP-gephyrin clusters at higher magnification; scale bar, 4 μm). (**d**) FRAP images of single GFP-gephyrin clusters in the CA1 stratum radiatum after bleaching at 0 min. Series of images taken from a non-stimulated control culture and a culture 2–4 h after rMS. (**e**,**f**) Quantitative evaluation of FRAP experiments. (**e**) Accelerated GFP-gephyrin FRAP is observed following rMS *in vitro*. (**f**) Group data for averaged 70–80 min FRAP values in non-stimulated and stimulated slice cultures (control, *n*=53 clusters from eight cultures; rMS, *n*=35 clusters from six cultures; 5–12 clusters bleached per culture; Kruskal–Wallis test followed by Dunn's *post hoc* test; one data point in **f** outside the axis limits; pooled non-stimulated control data). Individual data points are indicated by grey dots. Values represent mean±s.e.m. (*P<0.05; ***P<0.001; ns, not significant differences).

Accordingly, we tested whether molecular changes of inhibitory postsynapses accompany the rMS-induced functional changes in mIPSC amplitudes (Fig. 3). Slice cultures were immunostained for gephyrin, that is, the major postsynaptic scaffolding protein to which synaptic GABA_A_Rs anchor^14^,^15^,^16^ (Fig. 3a). Previous work demonstrated a close correlation between inhibitory synaptic strength, that is, mIPSC amplitudes and the size and stability of gephyrin clusters in CA1 stratum radiatum of entorhinohippocampal slice cultures^17^. Indeed, 3 h after rMS a significant reduction in gephyrin cluster sizes and numbers was observed in the stratum radiatum (Fig. 3b), indicating that rMS *in vitro* induces coordinated structural and functional changes of inhibitory postsynaptic sites.

### rMS has no major effect on gephyrin expression

To test whether the observed changes in gephyrin cluster properties reflect rMS-induced changes in gephyrin expression, tissue mechanically isolated from slice cultures containing the CA1 region was subjected to western blot and quantitative polymerase chain reaction (qPCR) analyses (Supplementary Fig. 2). In these experiments no significant difference was observed between the two groups (stimulated versus non-stimulated cultures), suggesting that rMS-induced changes in gephyrin clusters (at 3 h pms) cannot be trivially explained by a strong reduction in the expression of total gephyrin protein or mRNA.

### rMS destabilizes GFP-gephyrin clusters

We then hypothesized that rMS-induced changes in gephyrin turn-over could explain our findings. Hence, the effects of rMS on dynamic properties of gephyrin clusters were assessed in slice cultures prepared from *Thy1-GFP/gephyrin* mice^17^. In these mice gephyrin tagged with enhanced green fluorescent protein (GFP) is expressed in a subset of principal neurons (Fig. 3c)^22^. To test for changes in the exchange rate of gephyrin, we determined fluorescence recovery after photobleaching (FRAP) of GFP-gephyrin clusters in the stratum radiatum of rMS-treated cultures versus non-stimulated control cultures^17^. After a 15-min baseline recording, individual GFP-gephyrin clusters were photobleached (<5% of initial fluorescence intensity). The recovery, that is, reappearance of GFP-fluorescence was then followed for 80 min at 5-min intervals (Fig. 3d). In non-stimulated control cultures 30% of the averaged pre-bleach GFP-gephyrin fluorescence recovered within ∼40 min. In contrast, following rMS 30% recovery was reached already ∼7.5 min after the bleach, and after 80 min >60% of the pre-bleach signal was detected (Fig. 3e,f). These experiments disclosed a significantly faster recovery of GFP-gephyrin clusters 2–4 h following rMS *in vitro* (Fig. 3f; for averaged 70–80 min values; pooled non-stimulated control data). Taken together, our FRAP experiments showed that the re-incorporation of GFP-gephyrin into individual gephyrin clusters is accelerated after rMS. Since GFP-gephyrin clusters of comparable pre-bleach intensity were assessed in these experiments (control: 1.0±0.02, *n*=53 clusters from eight cultures; rMS: 1.1±0.03, *n*=35 clusters from six cultures; values normalized to control; *P*=0.15; Mann–Whitney test), we attribute this acceleration to an increased turnover, that is, rMS-induced destabilization of postsynaptic gephyrin scaffolds^17^ 2–4 h following magnetic stimulation.

### rMS reduces size and number of GABA_A_Rα2 clusters

**Figure 4:**
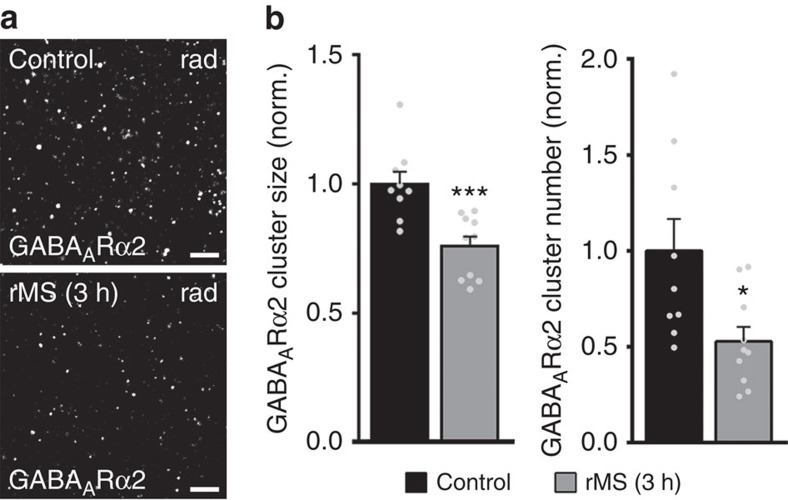
rMS induces a reduction in GABA_A_ receptor α2 clusters. (**a**) Stimulated and non-stimulated cultures were stained for GABA_A_ receptor subunit α2 (GABA_A_Rα2), which anchors GABA_A_Rs to postsynaptic gephyrin scaffolds. Cluster sizes and numbers were assessed in the CA1 stratum radiatum (rad). Scale bar, 3 μm. (**b**) rMS induces a decrease in the mean GABA_A_Rα2 cluster size and number. Values normalized to non-stimulated control cultures (control, *n*=9 cultures; rMS, *n*=10 cultures; averaged data from three visual fields per culture; Mann–Whitney test). Individual data points are indicated by grey dots. Values represent mean±s.e.m. (*P<0.05; ***P≤0.001; ns, not significant differences).

To link the described changes in gephyrin cluster properties to our electrophysiological recordings, we stained stimulated and non-stimulated slice cultures for GABA_A_R subunit α2, which anchor synaptic GABA_A_Rs to gephyrin^23^ (Fig. 4a). Indeed, a significant reduction in GABA_A_Rα2 cluster sizes and numbers was observed in the stratum radiatum 3 h after stimulation in these experiments (Fig. 4b).

### Paired recordings disclose rMS effects on synaptic inhibition

**Figure 5:**
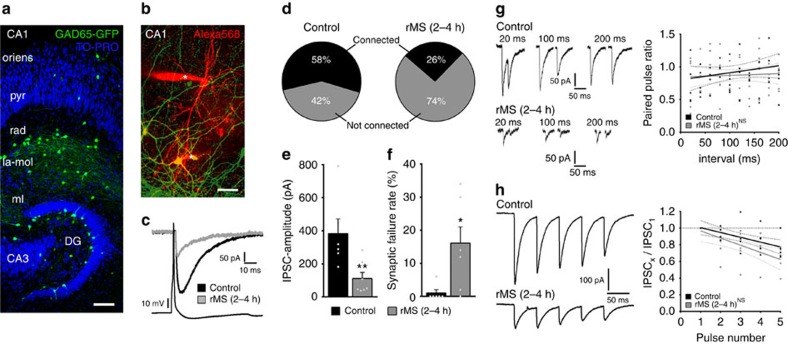
Paired recordings disclose changes in inhibitory synaptic strength, efficacy and connectivity following rMS. (**a**) Overview of a slice culture prepared from *GAD65-GFP* mice. Note GFP-expressing interneurons in the stratum radiatum (rad) and lacunosum moleculare (la-mol) of CA1. Occasionally, GFP-expressing interneurons are found in the stratum pyramidale (pyr) and in stratum oriens (oriens). Blue, TO-PRO nuclear stain. Scale bar, 100 μm. (**b**) Example of simultaneously patched neurons filled with Alexa568 (10 μM). GFP-expressing interneurons are patched in rad or at the border between rad and la-mol. Asterisks indicate positions of the tips of Alexa568-filled patch pipettes. Scale bar, 50 μm. (**c**) Averaged responses of successfully evoked time-locked postsynaptic currents from CA1 neurons in control (black trace) and stimulated slice cultures (grey trace). Up to 50 action potentials (45±3) were induced at 0.1 Hz in presynaptic GFP-expressing interneurons while recording from postsynaptic CA1 neurons. (**d**–**f**) A decrease in the percentage of connected pairs and evoked GABA_A_R-mediated IPSC amplitudes was observed, while the percentage of transmission failure rate was markedly increased after rMS (2–4 h pms; control; *n*=6 connected pairs in four cultures, one value (1,196 pA with 0% failure rate) excluded from analysis; 7 of 12 probed pairs connected; rMS, *n*=7 connected pairs in four cultures; 7 of 27 probed pairs connected; Mann–Whitney test). (**g**) Sample traces illustrating paired-pulse protocols at different interpulse intervals (left). No significant difference between non-stimulated and stimulated cultures in paired-pulse depression is observed (control, *n*=5 cells in four cultures; rMS (2–4 h), *n*=5 cells in four cultures, linear regression fit, Kruskal–Wallis test followed by Dunn's *post hoc* test; six data points outside the axis limits). (**h**) Sample traces illustrating short-term plasticity (five action potentials at 20 Hz). The amplitude of each consecutive inhibitory postsynaptic current was normalized to the amplitude of the first response. No significant difference between non-stimulated and stimulated cultures (control, *n*=5 cells in four cultures; rMS (2–4 h), *n*=5 cells in four cultures, linear regression fit, Kruskal–Wallis test followed by Dunn's *post hoc* test; one data points outside the axis limits). Individual data points are indicated by grey dots. Values represent mean±s.e.m. (*P<0.05; *P<0.01; ns, not significant differences).

To further characterize rMS-induced changes in inhibitory synaptic transmission at the level of individual connected neurons, paired recordings were carried out. In this set of experiments, slice cultures prepared from *GAD65-GFP* mice^24^ were used (Fig. 5). Interneurons mainly projecting their axons on dendrites of CA1 neurons can be readily identified by the GFP signal in these preparations^22^ (Fig. 5a). GFP-expressing interneurons (in the stratum radiatum or at the border to the stratum lacunosum moleculare) and CA1 pyramidal neurons were patched at the same time, and the connectivity between pairs of neurons was probed in stimulated and non-stimulated cultures (Fig. 5b). Neurons were considered connected if >5% of presynaptic action potentials evoked time-locked postsynaptic inward current responses (Fig. 5c; up to 50 action potentials induced at 0.1 Hz). In non-stimulated control cultures the probability to find connected pairs was ∼60% (Fig. 5d). Following rMS, a marked decrease (down to ∼25%) in the probability to find connected neurons was observed (Fig. 5d). In addition, the mean amplitude of successfully evoked IPSCs in response to single presynaptic action potentials was significantly decreased following rMS (Fig. 5e). Interestingly, a marked increase in the percentage of action potentials not successfully evoking postsynaptic current responses in connected pairs was detected 2–4 h after rMS (Fig. 5f; synaptic failure rate). Because paired-pulse and short-term plasticity were not significantly changed after rMS (Fig. 5g,h), we attribute the observed changes in failure rates to a decrease in the number of functional synapses between inhibitory interneurons and CA1 neurons, rather than to changes in the presynaptic release probability. This suggestion is in line with our findings on reduced gephyrin and GABA_A_Rα2 cluster numbers in the stratum radiatum after rMS (Figs 3 and 4), while mIPSC frequencies were not significantly reduced (Fig. 1g,h). Hence, although rMS-induced presynaptic changes cannot be excluded, these experiments confirmed that rMS leads to profound changes in the connectivity of inhibitory networks, that is, fewer, less efficient and weaker inhibitory synapses on CA1 pyramidal neurons.

### VGSC activity is required for rMS-induced plasticity

Which signals mediate the rMS-induced reduction in inhibitory synaptic transmission? Because rMS is expected to depolarize neurons and to induce action potentials^25^, we first tested whether the activation of VGSCs is required for the rMS-induced structural and functional remodelling of inhibitory postsynapses (Table 1; TTX). To this end, slice cultures were stimulated in the presence of TTX (2 μM; washed out immediately after stimulation)^6^ and mIPSC recordings, gephyrin immunostainings, GFP-gephyrin FRAP experiments and GABA_A_Rα2 immunostainings were repeated (*c.f.*, Figs 1, 3 and 4). Indeed, in these experiments the effects of rMS *in vitro* on structural and functional properties of inhibitory postsynapses were completely blocked. We conclude that VGSC activity is required for rMS-induced plasticity of inhibitory postsynaptic sites.

### NMDAR and L-VGCC activities mediate rMS-induced plasticity

Considering the previously proposed Ca^2+^-dependent tuning of inhibitory synapses, which accompanies LTP of excitatory synapses^19^ and our earlier findings on the role of NMDARs and L-VGCCs in rMS-induced potentiation of excitatory synapses^5^,^6^, we next tested whether pharmacological inhibition of NMDARs with AP5 (50 μM) or blocking L-VGCCs with Nifedipin (20 μM) hampers rMS-induced plasticity of inhibitory synapses (Table 1; AP5 and Nifedipin; inhibitors were applied only during rMS and washed out immediately after the end of stimulation). Except for a reduction in gephyrin cluster numbers in AP5-treated-stimulated cultures, we observed that mIPSC amplitudes, gephyrin clusters, GFP-gephyrin FRAP and GABA_A_Rα2 cluster sizes and numbers in the stratum radiatum were comparable in stimulated and non-stimulated slice cultures. We conclude from these experiments that rMS induces Ca^2+^-dependent tuning of inhibitory inputs, that is, a reduction in inhibitory synaptic strength, which involves the activation of NMDARs and L-VGCCs during stimulation.

### Calcineurin mediates rMS-induced plasticity

To test for possible downstream signalling pathways, we next blocked Ca^2+^-dependent, non-lysosomal cysteine proteases (calpain) or calcineurin protein phosphatases. Previous work showed that the degradation/destabilization of gephyrin clusters can be mediated by these Ca^2+^-dependent signalling pathways^18^,^19^. Accordingly, cultures were stimulated in presence of MDL-28170 (50 μM) to block calpain or Cyclosporin A (2 μM) to block calcineurin. In these experiments, pharmacologic inhibition of enzymatic activity was continued after stimulation. We found that MDL-28170 did not block the rMS-induced weakening of inhibitory inputs (Table 1; MDL-28170; changes in GABA_A_Rα2 cluster numbers did not reach the level of significance), but Cyclosporin A prevented the reduction in mIPSC amplitude, the remodelling of gephyrin clusters, the destabilization of GFP-gephyrin clusters and reduction in GABA_A_Rα2 cluster sizes and numbers following rMS *in vitro* (Table 1; Cyclosporin A; a slight increase in gephyrin cluster sizes was observed in Cyclosporin A-treated-stimulated cultures). Taken together, these experiments suggest that rMS remodels inhibitory synapses in a calcineurin-dependent manner.

### rMS does not affect all inhibitory synapse equally

**Figure 6:**
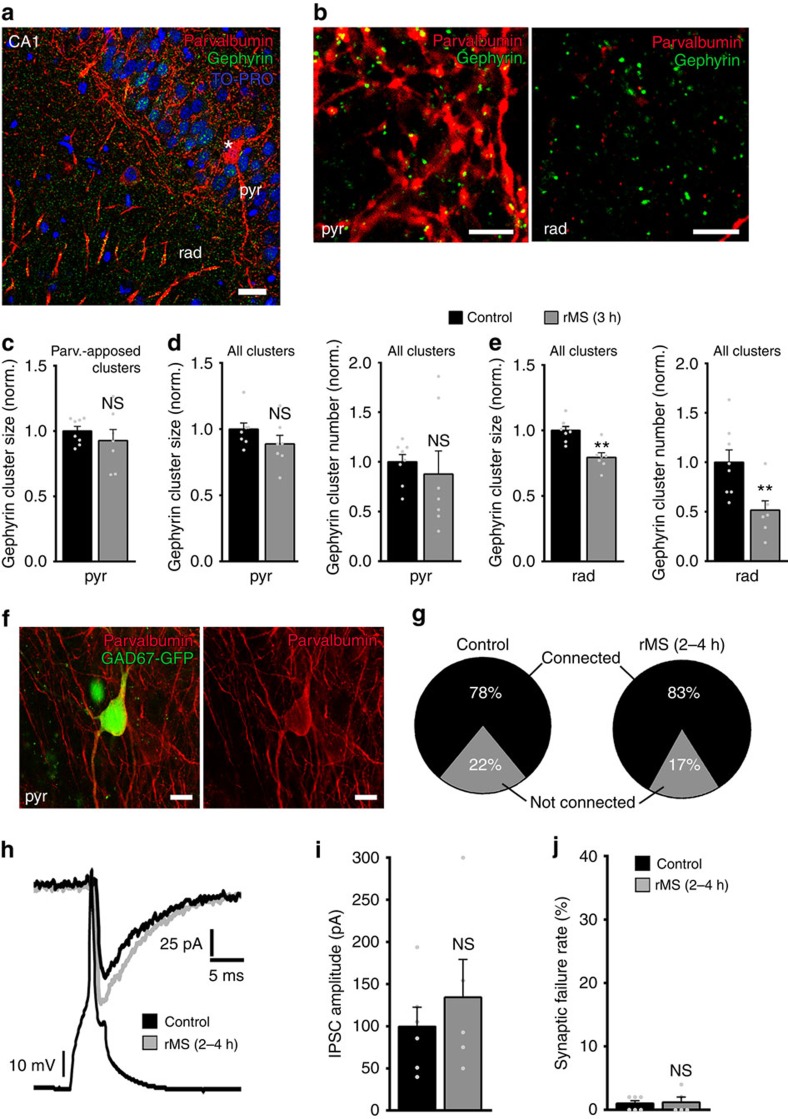
rMS does not affect somatic inhibition. (**a**,**b**) Slice cultures stained for gephyrin (green) and parvalbumin (red). Note the parvalbumin-positive interneuron (asterisk in **a**) in pyr. The axons of these neurons arborize mainly within the pyramidal cell layer (**b**; rad). The size of gephyrin clusters associated with parvalbumin-positive presynaptic boutons was assessed in pyr. Blue, TO-PRO nuclear stain. Scale bar in **a**, 20 μm, in **b**, 4 μm. (**c**) No significant difference in parvalbumin-associated gephyrin cluster sizes is observed in pyr between stimulated and non-stimulated slice cultures 3 h after rMS (control, *n*=8 cultures; rMS, *n*=7 cultures; averaged data from three visual fields per culture; Mann–Whitney test). (**d**,**e**) The mean size and number of all gephyrin clusters are not significantly different in pyr. In the rad a significant reduction in gephyrin cluster sizes and numbers is observed after stimulation in the same set of cultures (control, *n*=8 cultures; rMS, *n*=7 cultures; averaged data from three visual fields per culture; Mann–Whitney test). (**f**) Paired recordings of parvalbumin-positive interneurons and CA1 pyramidal neurons were carried out in slice cultures prepared from *GAD67-GFP* mice. Parvalbumin-positive interneurons in pyr can be readily identified by the GFP signal in these preparations. Scale bar, 10 μm. (**g**–**j**) No major changes in inhibitory synaptic strength, efficacy and connectivity are observed in these experiments (control; *n*=6 connected pairs in four cultures, one connected pair excluded from analysis since series resistance changed significantly during recordings and reached ⩾30 MΩ; seven of nine probed pairs connected; rMS, *n*=5 connected pairs in three cultures; five of six probed pairs connected; Mann–Whitney test). Individual data points are indicated by grey dots. Values represent mean±s.e.m. (**P<0.01; ns, not significant differences).

Since our recent work revealed that rMS *in vitro* acts on specific subsets of excitatory inputs^6^, and because spatially separate inhibitory inputs are considered to mediate diverse neuronal functions^9^,^26^,^27^, we next tested whether rMS *in vitro* acts differentially on distinct subsets of inhibitory postsynapses. At this point, we focused on comparing somatic versus dendritic inhibition. First, slice cultures were double-stained for gephyrin and parvalbumin, which is a marker for inhibitory interneurons that project their axons mainly within the pyramidal cell layer, that is, the layer in which the cell bodies of CA1 pyramidal neurons are located (Fig. 6a). In these experiments, we assessed gephyrin clusters that were associated with parvalbumin-positive structures in the stratum pyramidale (Fig. 6b) and found no change in cluster sizes at 3 h following rMS (Fig. 6c). Analysis of all clusters in the stratum pyramidale provided similar results (Fig. 6d), while in the same set of cultures a reduction in the mean gephyrin cluster size and number was observed in the stratum radiatum (Fig. 6e; all clusters analysed). These experiments indicate that rMS may not affect somatic inhibition while reducing dendritic inhibition.

### rMS does not affect somatic inhibition

To verify these results, paired recordings of parvalbumin-positive interneurons and CA1 pyramidal neurons were employed, similar to the experiments described in Fig. 5. To readily identify parvalbumin-expressing interneurons, slice cultures prepared from *GAD67-GFP*^28^-transgenic mice were used. After confirming the identity of GFP-expressing neurons in the CA1 stratum pyramidale by immunostaining for parvalbumin (Fig. 6f; different set of cultures), pairs of neurons were recorded to determine connectivity, as well as strength and efficacy of CA1 somatic inhibitory neurotransmission (Fig. 6g–j). At the end of the experiments, the fast spiking property of the patched GFP-positive interneuron was confirmed (Supplementary Fig. 3). No significant difference was observed between stimulated and non-stimulated slice cultures in these experiments (Fig. 6i,j; paired-pulse protocol and short-term plasticity given in Supplementary Fig. 3). Of note, in the same set of recorded CA1 neurons a significant reduction in the mean amplitude of spontaneous IPSCs was detected (sIPSC amplitude, control: 18.8±1.0 pA, *n*=8 neurons from four cultures; rMS: 14.5±1.3 pA, *n*=9 neurons from three cultures; *P*<0.05; sIPSC frequency, control: 6.3±1.3 Hz, *n*=8; rMS: 5.2±0.7 Hz, *n*=9; *P*=0.66; Mann–Whitney test). We conclude from these experiments that 10-Hz rMS *in vitro* does not exert its major effects by changing somatic inhibition.

### GABA uncaging reveals rMS effects on dendritic inhibition

**Figure 7:**
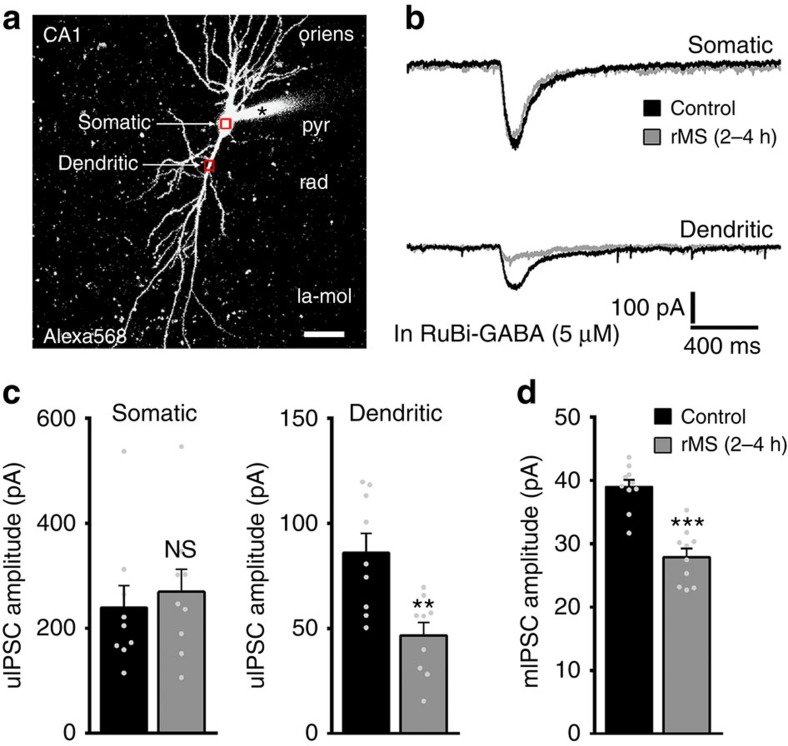
Flash photolysis of caged GABA discloses that rMS affects predominantly dendritic inhibition. (**a**) Flash photolysis of RuBi-GABA was employed to separately assess dendritic and somatic inhibition in the same set of patched CA1 pyramidal neurons (filled with Alexa568 before recordings; la-mol). Scale bar, 30 μm. (**b**) Representative averaged traces (five consecutive laser-light pulses at 0.1 Hz in each region) showing RuBi-GABA-uncaging-evoked somatic and dendritic responses recorded from non-stimulated control cultures and cultures between 2 and 4 h after rMS. (**c**,**d**) Group data of uncaging-evoked inhibitory postsynaptic current (uIPSC) amplitudes (*n*=9 neurons per group; five cultures each; Mann–Whitney test). In the same set of cultures mIPSC recordings confirmed a significant reduction in the mean mIPSC amplitude of CA1 pyramidal neurons (note: internal solution contained higher [Cl^−^] than in Fig. 1h; control, *n*=10 neurons; rMS, *n*=10 neurons from four cultures each; Mann–Whitney test). Individual data points are indicated by grey dots. Values represent mean±s.e.m. (**P<0.01; ***P<0.001; ns, not significant differences).

To provide further support for the differential effects of rMS on inhibitory postsynapses, rMS-induced functional changes in somatic versus dendritic inhibition were determined in the same set of neurons using flash photolysis of caged-GABA (Fig. 7; ‘GABA-uncaging experiments'). This technique enabled us to separately stimulate somatic versus dendritic GABA_A_Rs while recording uncaging-evoked IPSCs from the soma of individual CA1 pyramidal neurons (Fig. 7a,b). Care was taken to accurately position the laser beam for locally releasing GABA to target either the soma or proximal part of the apical principal dendrite of the Alexa-filled postsynaptic neuron (Fig. 7a). For each recorded neuron, Ruthenium-bipyridine-triphenylphosphine caged GABA (RuBi-GABA)-uncaging-evoked inward current responses to five consecutive laser-light pulses at 0.1 Hz in each region (somatic versus dendritic) were averaged (Fig. 7b). The uncaging responses were blocked by the GABA_A_R inhibitor SR95531 and did not occur when the laser beam was directed on the neurophil in the neighbourhood of the patched CA1 pyramidal neurons (that is, >10 μm away from the soma or closest dendrite)^29^. Responses to RuBi-GABA uncaging at somatic locations were not significantly different in the two groups, whereas dendritic GABA responses were markedly decreased in the rMS group (Fig. 7c). Since these experiments were performed in the presence of TTX, AP5 and CNQX, we were also able to record mIPSC events from a different set of neurons in this round of experiments (Fig. 7d). Thus, we verified once more that the mean mIPSC amplitude is reduced 2–4 h after rMS (Fig. 7d; internal solution containing higher [Cl^−^] compared with Fig. 1). Together with our paired recordings and the gephyrin immunostaining (Figs 5 and 6), we conclude that 10-Hz rMS *in vitro* predominantly affects dendritic (but not somatic) GABA_A_R-mediated synaptic inhibition.

### rTMS induces remodelling of clustered gephyrin *in vivo*

**Figure 8:**
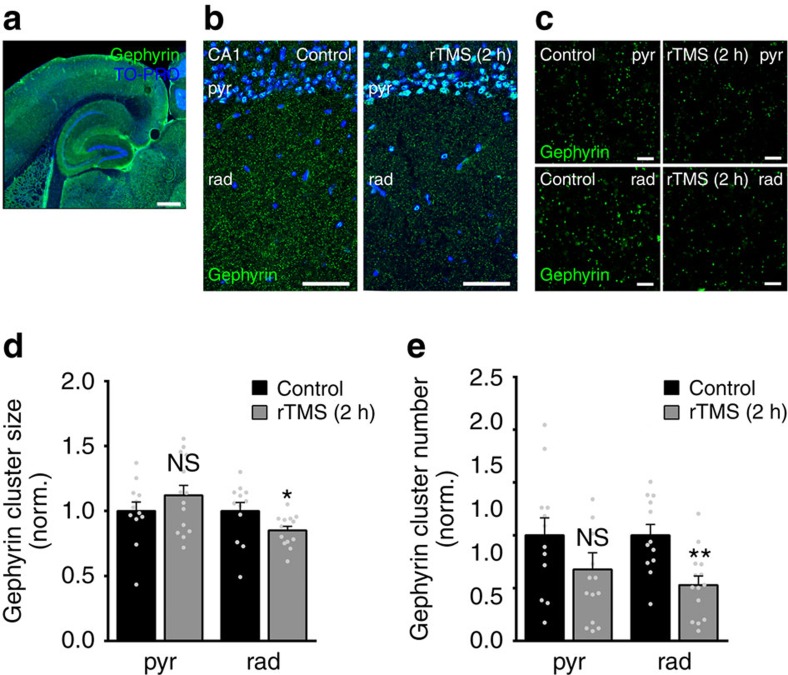
TMS induces changes in gephyrin clusters *in vivo*. (**a**–**c**) Examples of horizontal brain slices containing the hippocampus stained for gephyrin from non-stimulated anaesthetized 3-month-old male mice and 2 h after 10-Hz rTMS. Experimental conditions were adapted to the *in vitro* experiments. An increase in gephyrin immunostaining co-localizing with TO-PRO nuclear stain (blue) was noted. Scale bar in **a**, 300 μm, in **b**, 50 μm, in **c**, 4 μm. (**d**,**e**) A significant reduction in gephyrin cluster sizes and numbers was observed in rad but not in pyr 2 h after stimulation (control, *n*=12 hippocampi from six animals; rTMS, *n*=14 hippocampi from seven animals; Mann–Whitney test). Individual data points are indicated by grey dots. Values represent mean±s.e.m. (*P<0.05; **P<0.01; ns, not significant differences).

As a proof-of-principle, we finally tested whether rMS affects gephyrin scaffolds in the intact animal. In this series of experiments, 10-Hz rTMS of anaesthetized 3-month-old male wild-type mice was employed (Fig. 8). The experimental conditions were adapted to our slice culture experiments: (1) orientation of the coil was matched with respect to stimulation of the hippocampus in horizontal slices, that is, portions of the ventral hippocampus used for our slice preparations. (2) The same 10-Hz protocol was employed (at 90% motor threshold; ∼60% of maximum stimulator output, MSO^30^). (3) Animals were killed 2 h after stimulation and horizontal brain slices containing the hippocampus were stained for gephyrin (Fig. 8a–c). (4) Gephyrin cluster sizes and cluster numbers were assessed in the CA1 stratum pyramidale and stratum radiatum (Fig. 8b,c). Indeed, a significant reduction in gephyrin cluster sizes and numbers was observed in the stratum radiatum but not the stratum pyramidale 2 h after stimulation (Fig. 8d,e). Together, these experiments demonstrate that 10-Hz rMS is a potent tool to induce the remodelling of inhibitory postsynapses, that is, gephyrin scaffolds *in vitro* and *in vivo*.

## Discussion

Alterations in E/I balance and disturbed cortical homeostasis^31^,^32^,^33^ have been suggested to cause behavioural and cognitive dysfunction in many brain diseases, such as schizophrenia^11^,^12^, autism^10^,^13^ and panic disorders^34^. In these disease contexts, the diagnostic and therapeutic potentials of non-invasive brain stimulation techniques have been extensively studied^1^. Still, the cellular and molecular mechanisms of rTMS-mediated neural plasticity and hence rTMS-based therapies remain incompletely understood^2^,^3^,^4^. While it has been proposed that LTP/LTD-like plasticity of excitatory synapses underlies rTMS effects on cortical excitability^35^, less attention has been dedicated to rTMS-induced changes in inhibitory neurotransmission^36^,^37^,^38^. The present study demonstrates that 10-Hz rMS induces structural and functional plasticity of inhibitory synapses. These changes depend on the activation of VGSCs, L-VGCCs and NMDARs and are not observed when calcineurin protein phosphatases are pharmacologically blocked. Since dendritic inhibition appeared to be mainly affected in our experiments, we conclude that 10-Hz rMS acts through the Ca^2+^-dependent remodelling of specific, that is, dendritic inhibitory synapses of principal neurons. These results disclose a mechanism how rTMS could modulate E/I balance and connectivity in neuronal networks.

We regard it as one of the major findings of our study that rMS is able to induce the remodelling of gephyrin scaffolds. Gephyrin is thought to form a hexagonal lattice beneath the postsynaptic membrane^39^,^40^,^41^ and to determine synaptic efficacy by immobilizing GABA_A_Rs and glycine receptors at synaptic sites^42^,^43^,^44^,^45^,^46^,^47^,^48^. Both the receptors and gephyrin are continuously exchanged because of dissociation from and re-association to postsynaptic sites^49^,^50^. The size and properties of gephyrin clusters are regulated by neuronal activity^17^,^19^ and phosphorylation^39^,^51^, and depend on interactions with the cytoskeleton^52^. As discussed elsewhere^14^, synaptic signalling pathways triggered by Ca^2+^ influx^53^ are considered to regulate inhibitory postsynaptic scaffold dynamics and thereby to control GABA_A_R recruitment and internalization rates. The results of the present study are in line with this suggestion, since inhibition of NMDARs and L-VGCCs abolished the rMS-induced reduction in gephyrin cluster size and stability in the stratum radiatum. While our MDL-28170 experiments did not support a role of calpain-mediated gephyrin degradation^18^, we were able to provide evidence that calcineurin-regulated phosphorylation/dephosphorylation reactions are involved in rMS-induced remodelling of inhibitory postsynapses (see also refs 39, 53, 54). Hence, it is likely that rMS acts on inhibitory neurotransmission by modulating the Ca^2+^/calcineurin-dependent gephyrin oligomerization/dissociation rather than gephyrin synthesis/degradation; a suggestion that is also supported by our qPCR and western blot results on unaltered gephyrin mRNA and protein levels 3 h after rMS *in vitro*. Whether the rMS-induced destabilization of gephyrin scaffolds, the reduction in gephyrin cluster sizes and gephyrin cluster numbers resemble distinct mechanisms that share overlapping signalling pathways, or different stages of the same process needs to be determined (*c.f.*, differential effect of NMDAR inhibition on rMS-induced gephyrin cluster size/stability and numbers; Table 1). Regardless of these considerations, our study provides robust experimental evidence that rMS is a suitable tool to induce the remodelling of gephyrin scaffolds (both *in vitro* and *in vivo)*. In light of the fact that rare exonic deletions implicate gephyrin in risk for autism, schizophrenia and seizures^55^, we propose that a better understanding of rMS-mediated changes in gephyrin-dependent inhibitory synaptic plasticity (under physiological and pathological conditions) may provide the perspective to transfer basic science knowledge into clinical practice and eventually devise new diagnostic and therapeutic strategies using non-invasive brain stimulation techniques.

Using immunostainings for gephyrin, GABA-uncaging experiments and paired recordings, we demonstrate that rMS changes the strength, efficacy and connectivity of dendritic inhibition, while having no major impact on somatic inhibition. The precise mechanisms through which rMS exerts its effects predominantly on dendritic (but not somatic) inhibition warrant further investigation. For example, differential effects of the induced electric field on somatic versus dendritic compartments, on specific subtypes of interneurons or glial cells are possible. Alternatively, intracellular calcium stores and the distribution of distinct channels, receptor (subunits) and enzymes may play a role. It is also possible that inhibitory synapses in the soma are less susceptible to Ca^2+^-mediated remodelling. Thus, our 10-Hz rMS protocol may not induce somatic Ca^2+^ levels high enough to trigger plastic changes of inhibitory postsynapses in this compartment. The requirement of both NMDAR and L-VGCC activity during stimulation indicates that a synergistic activation of these Ca^2+^ entry sites is required for the local remodelling of inhibitory postsynapses to occur after rMS. Since pyramidal cells of the cortex do not carry many asymmetric, that is, NMDAR-containing excitatory synapses on their soma^56^, this synergy may simply not occur at the soma.

Although the functional consequences of rMS-induced changes in inhibition for network function and behaviour^57^,^58^,^59^ remain unknown, it is interesting to hypothesize that rTMS may assert its beneficial effects seen in the context of neuropsychiatric diseases by modulating specific molecular and functional aspects of inhibition in cortical networks, for example, dendritic inhibition. This suggestion is supported by recent experimental evidence that indicates that the recruitment of disinhibitory microcircuits plays an important role in cortical plasticity^7^. Hence, it is conceivable that rMS-induced disinhibition could prime cortical networks for the expression of subsequent (experience-dependent) plasticity. However, at this point we have to concede that we do not know enough about the temporal sequence and molecular interdependency of rMS-induced disinhibition and other forms of plasticity, except that both rMS-induced LTP of excitatory synapses^5^,^6^ and LTD of inhibition (this study) require the activation of VGSCs, L-VGCCs and NMDARs during stimulation.

It will now be important to test whether the outcome of rMS on synaptic plasticity depends on the architecture and state of the stimulated network or the specific stimulation protocol employed. Differential effects of rMS on specific inhibitory (and excitatory) synapses might be observed when stimulus intensities, frequencies, total number of applied magnetic pulses and the orientation of the stimulated tissue within the electromagnetic field are modified^60^, or under conditions in which E/I balance is pharmacologically or genetically altered (for example, to mimic specific pathological brain conditions; see also ref. 30, who describe differential outcomes of rTMS in anaesthetized and awake animals). Nevertheless, by carefully adapting our stimulation parameters to the *in vivo* situation we could verify that rMS-induced changes in gephyrin scaffolds are also seen in the intact animal situation. We are confident that future *in vitro* and *in vivo* studies of the hippocampus and other brain regions, possibly in combination with computational approaches, which will help to explore the enormous parameter space, will provide novel insights on the dose-, orientation- and state dependency of rMS-mediated neural plasticity and its outcome on E/I balance under physiological and pathological conditions. These studies may support the transfer of basic science knowledge on neural plasticity into clinical practice and could thereby also help addressing some of the important questions regarding inter- and intraindividual variabilities of rTMS effects in human subjects^2^,^61^.

## Methods

### Ethics statement

Mice were maintained in a 12-h light/dark cycle with food and water available *ad libitum*. Every effort was made to minimize distress and pain of animals. All experimental procedures were performed according to the German animal welfare legislation and approved by the appropriate animal welfare committee and the animal welfare officer of Goethe-University Frankfurt, Faculty of Medicine.

### Preparation of slice cultures

Entorhinohippocampal slice cultures were prepared on postnatal days 4–5 from *C57BL/6J*, *Thy1-GFP/gephyrin* (heterozygous for GFP-Gephyrin)^17^, *GAD65-GFP*^24^ and *GAD67-GFP*^28^ (heterozygous for GFP; obtained from Jackson Laboratories, USA) mice of either sex^17^. Cultivation medium contained 50% (v/v) MEM, 25% (v/v) basal medium eagle, 25% (v/v) heat-inactivated normal horse serum (NHS), 25 mM HEPES buffer solution, 0.15% (w/v) bicarbonate, 0.65% (w/v) glucose, 0.1 mg ml^−1^ streptomycin, 100 U ml^−1^ penicillin and 2 mM glutamax. The pH was adjusted to 7.3 and the medium was replaced three times per week. All slice cultures were allowed to mature for at least 18 days in humidified atmosphere with 5% CO_2_ at 35 °C. Cultures prepared from at least three independent litters were used in each biological experiment.

### rMS *in vitro*

Slice cultures (⩾18 days *in vitro*) were transferred to a non-temperature-controlled chamber, that is, a 30-mm Petri dish containing standard extracellular solution (129 mM NaCl, 4 mM KCl, 1 mM MgCl_2_, 2 mM CaCl_2_, 4.2 mM glucose, 10 mM HEPES, 0.1 mg ml^−1^ streptomycin, 100 U ml^−1^ penicillin, pH 7.4 with KOH; preheated to 35 °C; osmolarity adjusted with sucrose to match cultivation medium). Cultures were stimulated in the absence of any wires and electrodes using a Magstim Rapid stimulator with a biphasic current waveform, using two booster modules (Magstim Company, UK) and connected to a standard 70-mm outer wing diameter double, that is, figure-of-eight coil (Magstim Company). Cultures were positioned 1 cm under the centre of the coil (that is, junction of the two wings) and stimulated with a protocol consisting of nine trains of 100 pulses each at 10 Hz with an intertrain interval of 30 s (at 50% of MSO). Orientation of cultures was such that the induced electric field within the tissue was approximately parallel to the dendritic tree of CA1 pyramidal neurons. In some experiments, TTX (2 μM), AP5 (50 μM) or Nifedipin (20 μM) were used to block VGSCs, NMDARs or L-VGCCs during stimulation, respectively. Drugs were immediately washed out after stimulation. Cultures were kept in the incubator for at least 2 h after stimulation before experimental assessment. Cyclosporin A (2 μM) or MDL-28170 (50 μM) were used to block the Ca^2+^-dependent phosphatase calcineurin or the Ca^2+^-dependent protease calpain, respectively. Age- and time-matched control cultures were not stimulated, but otherwise treated identical to stimulated cultures.

### Modelling the electric field *in vitro*

The electric field induced by rMS *in vitro* was described based on a magnetic dipole model of the Magstim 70-mm figure-of-eight coil^62^. A finite element method model was created with the dimensions of the bath and tissue adapted to the *in vitro* setting. The coil was positioned 1 cm above the tissue. Electrical conductivities were 0.3 S m^−1^ for the tissue and 1.4 S m^−1^ for the bath solution^63^,^64^,^65^. The rate of change of the coil current was 115.1 A μs^−1^, which corresponds to 50% MSO in the experiments. The electric field was solved using a quasistatic approximation^66^ with the SimNibs toolbox^67^. The computed electric field (27.3±3.3 V m^−1^)^68^ in the tissue was nearly perfectly aligned along the coil axis with a mean absolute angular deviation of 4.3°. The effects of slight changes in coil-to-slice culture distance were estimated by comparing the induced electric field strengths for distances of 1, 1.1 and 1.2 cm. Under these conditions, differences of <2.5 V m^−1^ were determined.

### rTMS of anaesthetized mice

rTMS was carried out in 3-month-old male urethane-anaesthetized *C57BL/6J* mice (1.25 g kg^−1^, intraperitoneal; 0.125 g kg^−1^, subcutaneous). The head was placed under the centre of the coil and the orientation of the coil was optimized to mirror the *in vitro* setting, in which the hippocampus taken from horizontal brain slices is used for cultivation and subsequent rMS. Brain-to-coil distance was kept minimal while assuring contact-free stimulation. The motor threshold was determined before each experiment using single pulses at 0.2 Hz with increasing stimulator intensity (5% MSO steps, starting from 40% MSO). Repetitive stimulation was performed at 90% of motor threshold (corresponding to 59±1% MSO) using the same 10-Hz stimulation protocol described above. Control animals placed near the coil during stimulation were not stimulated but otherwise treated identically to stimulated animals. All animals were transferred back to their cages and held in anaesthesia for 2 h. Deeply anaesthetized mice were rapidly decapitated. Brains were removed and briefly washed in ice-cold PBS before embedding in Tissue-Tek freezing medium (Sakura Finitek). After 3 min in 2-methylbutan at −40 °C brains were stored at −20 °C.

### Immunostaining and imaging

Slice cultures were fixed in a solution of 4% (w/v) paraformaldehyde (PFA) in PBS (0.1 M, pH 7.4) and 4% (w/v) sucrose for 1 h, followed by 2% PFA and 30% sucrose in PBS overnight. Cryostat sections (30 μm) of fixed slice cultures were prepared and stained with antibodies against gephyrin (Synaptic Systems, clone mAb7a; 1:500), GABA_A_Rα2 (Santa Cruz Biotechnology, sc-7350; 1:500), GABA_A_Rα5 (Sigma-Aldrich, SAB-2701358; 1:500) and/or parvalbumin (Swant, PV-28; 1:200) following a modified protocol previously described^69^. Briefly, the sections were incubated for 1 h with 10% (v/v) normal goat serum (NGS) or NHS in 0.5% (v/v) Triton X-100 containing PBS to reduce unspecific staining and subsequently incubated for 48 h at 4 °C with the respective primary antibodies (in PBS with 10% NGS or NHS and 0.1% Triton X-100). Sections were washed and incubated for 3 h with appropriate Alexa488 or 568-labelled secondary antibodies (Invitrogen; 1:1,000, in PBS with 10% NGS or NHS, 0.1% Triton X-100). TO-PRO (Invitrogen) nuclear stain was used to visualize cytoarchitecture (1:5,000; in PBS for 10 min). The sections were washed, transferred on glass slides and mounted for visualization with anti-fading mounting medium.

Horizontal cryostat sections (35 μm) containing the ventral hippocampus were prepared from whole-mouse brains and immediately mounted on glass slides. Sections were fixed with 4% PFA/4% sucrose for 15 min. After washing with PBS, the slices were incubated for 1 h with 10% NGS in 0.5% Triton X-100 containing PBS to reduce unspecific staining and subsequently incubated overnight at 4 °C with mouse anti-gephyrin antibody (Synaptic Systems, clone mAb7a; 1:500, in PBS with 10% NGS and 0.1% Triton X-100). Sections were washed and incubated for 2 h with Alexa488-labelled goat anti-mouse antibody (Invitrogen; 1:500, 10% NGS, 0.1% Triton X-100). TO-PRO (Invitrogen) nuclear stain was used to visualize cytoarchitecture (1:5,000; in PBS for 10 min). Sections were washed again, transferred on glass slides and mounted for visualization with anti-fading mounting medium.

A Nikon Eclipse C1si laser-scanning microscope with a × 4 objective lens (numeric aperture (NA) 0.2, Nikon), a × 40 objective lens (NA 1.3, Nikon) and a × 60 oil-immersion objective lens (NA 1.4, Nikon) was used for confocal microscopy. Three visual fields per region of interest were imaged in each culture at high magnification. Detector gain and amplifier were set to obtain pixel intensities within a linear range.

### Fluorescence recovery after photobleaching

FRAP experiments were performed with a Zeiss LSM Exciter confocal microscope at 35 °C (bath solution contained 126 mM NaCl, 2.5 mM KCl, 26 mM NaHCO_3_, 1.25 mM NaH_2_PO_4_, 2 mM CaCl_2_, 2 mM MgCl_2_ and 10 mM glucose)^17^,^69^. Individual GFP-gephyrin clusters in the stratum radiatum of area CA1 were imaged with a × 40 water immersion objective lens (0.8 NA; Zeiss) and × 2 scan zoom. The pinhole diameter was set at 1 Airy Unit, and image stacks (seven images) were taken at an ideal Nyquest rate. Imaging parameters were optimized to minimize bleaching by the imaging procedure. Following a 15-min baseline registration (Δ*t*=5 min), selected GFP-gephyrin clusters in the middle plane of the stack were photobleached (<5% of initial fluorescence) using the bleaching function of the Zeiss Zen Software (Acousto Optic Tunable Filter, AOTF-controlled Argon laser 488 nm; 100% transmission; 100 bleach iterations) and FRAP was followed for 80 min (Δ*t*=5 min). Our earlier work revealed that in the CA1 stratum radiatum of entorhinohippocampal slice cultures ∼92% of the GFP-gephyrin clusters are synaptically localized, as revealed by co-immunolabelling for the presynaptic vesicular inhibitory amino-acid transporter^17^.

*Western blotting*. Isolated tissue containing the CA1 region was separated on 8% (w/v) SDS-polyacrylamide gels (30 μg protein per lane), transferred to nitrocellulose (Protran Whatman) and probed with anti-gephyrin (Synaptic Systems, 14711; 1:3,000) and anti-GAPDH (Calbiochem, CB1001; 1:10,000), followed by incubation with appropriate secondary antibodies (LI-COR, IRDye800 or IRDye680-conjugated antibodies; 1:10,000). Bound antibody was visualized using the Odyssey Infrared Imaging System (LICOR).

*RNA extraction and qPCR*. RNA from tissue containing the CA1 region was isolated using the RNeasy MicroPlus Kit (Qiagen). RNA integrity numbers (9.7±0.04) were determined using the Agilent 2100 Bioanalyzer system and Agilent RNA 6000 Pico Kit (Agilent Technologies, Germany). The High-Capacity cDNA Reverse Transcription Kit (Applied Biosystems, USA) was used to transcribe purified RNA into cDNA (all kits and assays used according to the manufacturers' instructions). The cDNA was amplified with the TaqManPreAmp Master Mix Kit (Applied Biosystems) using 5 μl PreAmp Master Mix (Applied Biosystems)+2.5 μl cDNA+2.5 μl Assay Mix (TaqMan Gene Expression (TM)-Assay (Gephyrin: Mm00556895_m1; GAPDH: 4352932E) from Applied Biosystems) with a standard amplification protocol (14 cycles: 95 °C for 15 s; 60 °C for 4 min). Amplified cDNAs were diluted 1:20 in ultrapure water and subjected to qPCR (StepOnePlus, Applied Biosystems) using a standard amplification programme (1 cycle of 50 °C for 2 min, 1 cycle of 95 °C for 10 min, 40 cycles of 95 °C for 15 s and 60 °C for 60 s; cutoff at 36 cycles; averaged *C*_t_ value was: 22.9±0.2 cycles).

### Whole-cell patch-clamp recordings

Whole-cell voltage-clamp recordings were carried out at 35 °C (two to five neurons per culture)^5^. The bath solution contained 126 mM NaCl, 2.5 mM KCl, 26 mM NaHCO_3_, 1.25 mM NaH_2_PO_4_, 2 mM CaCl_2_, 2 mM MgCl_2_ and 10 mM glucose. For mIPSC recordings, patch pipettes contained 40 mM CsCl, 90 mM K-gluconate, 1.8 mM NaCl, 1.7 mM MgCl_2_, 3.5 mM KCl, 0.05 mM EGTA, 2 mM ATP-Mg, 0.4 mM GTP-Na_2_, 10 mM PO-Creatine, 10 mM HEPES (pH=7.25 with KOH, 290 mOsm with sucrose), having a tip resistance of 4–6 MΩ. Alexa488 or Alexa568 (both 10 μM) was added to the internal solution in some experiments to visualize neuronal morphology before recordings. Neurons were recorded at holding potential −80 mV in the presence of 0.5 μM TTX, 10 μM AP5 and 10 μM CNQX. Series resistance was monitored in 2-min intervals, and recordings were discarded if the series resistance and leak current changed significantly and/or reached ⩾30 MΩ or ⩾350 pA, respectively. All electrophysiological recordings were performed from stimulated and non-stimulated slice cultures between 2 and 4 h after stimulation in a pseudorandomized manner to avoid acquisition bias.

### Tonic GABA_A_ receptor conductance

Patch pipettes contained 125 mM CsCl, 5 mM NaCl, 2 mM MgCl_2_, 2 mM Mg-ATP, 0.5 mM Na_2_-GTP, 0.1 mM EGTA and 10 mM HEPES (pH=7.33 with CsOH; 274 mOsm with sucrose). GABA_A_R currents were isolated using 0.5 μΜ TTX, 10 μΜ AP5 and 10 μΜ CNQX and in the bath solution, which enabled us to also detect mIPSC in the same set of recordings. The amplitude of tonic GABA_A_R current was determined by perfusing GABA (5 μΜ), followed by bicuculline-methiodide (50 μΜ) containing bath solution.

### Paired recordings

Simultaneous whole-cell patch-clamp recordings of neurons were carried out as described previously^5^,^29^. Slice cultures prepared from *GAD65-GFP*^24^ or *GAD67-GFP* mice were used to readily identify interneurons projecting their axons on dendrites or the soma of CA1 pyramidal neurons, respectively. Internal solution for presynaptic recordings of GFP-expressing interneurons contained 126 mM K-gluconate, 4 mM KCl, 4 mM ATP-Mg, 0.3 mM Na_2-_GTP, 10 mM PO-creatine, 10 mM HEPES and 0.1% biocytin (pH=7.25 with KOH, 290 mOsm with sucrose). CA1 pyramidal neurons in *GAD67-GFP* slices were recorded with low chloride solution (*c.f.*, mIPSC recordings) and in *GAD65-GFP* preparations with high chloride solution (*c.f.*, tonic inhibition). Action potentials were generated by 3-ms square current pulses (1 nA) elicited at 0.1 Hz (up to 50 pulses) while recording inhibitory postsynaptic currents from CA1 pyramidal neurons (recordings performed in the presence of AP5 and CNQX, both 10 μM; since these recordings were performed in the absence of TTX, we were also able to assess rMS-induced changes in spontaneous IPSCs). Paired-pulse kinetics of IPSCs were determined by inducing two presynaptic action potentials with increasing interpulse interval (20–200 ms, Δ*t*=20 ms at 0.1 Hz; at least four repetitions). For short-term plasticity, five action potentials were applied at 20 Hz (intersweep interval: 10 s; 30 repetitions).

### Flash photolysis of caged GABA

Alexa568 (10 μM) was added to the internal solution (125 mM CsCl, 5 mM NaCl, 2 mM MgCl_2_, 2 mM Mg-ATP, 0.5 mM Na_2_-GTP, 0.1 mM EGTA and 10 mM HEPES; pH=7.33 with CsOH; 274 mOsm with sucrose) to visualize cellular morphology before recordings. For local stimulation of GABA_A_Rs, CA1 pyramidal neurons were recorded in presence of RuBi-GABA (5 μM; Tocris Bioscience; in 0.5 μM TTX, 10 μM AP5 and 10 μM CNQX containing bath solution). The laser beam was focused on the maximal cross-sectional area of the soma or the principal apical dendrite within the stratum radiatum at a distance of ∼50 μm from the soma (Zeiss LSM Exciter confocal microscope equipped with a Zeiss × 40 water immersion objective lens; NA 0.8). A region of interest (30 × 30 pixels; ∼75 μm^2^) was selected (soma versus dendrite), and photolysis was performed using the bleaching function of the Zeiss Zen software (AOTF-controlled Argon laser 488 nm; 100% transmission; single bleach iteration, <1 ms duration; five times at 0.1 Hz per region), while recording inward current responses from the soma of CA1 pyramidal neurons in whole-cell voltage clamp configuration.

### Quantification and statistics

Analysis was performed by investigators blind to experimental conditions. Electrophysiological data were analysed using pClamp 10.2 (Axon Instruments) and MiniAnalysis (Synaptosoft) software. Overall, 150–350 mIPSC or sIPSC events were analysed per recorded neuron. Tonic inhibition was analysed by fitting Gaussian distributions to all point histograms for 10-s epochs of the recordings before and after the application of GABA and after the application of bicuculline-methiodide. Owing to the presence of mIPSCs, only the positive sites of the histograms were fitted under baseline conditions and after GABA application^70^. The differences between the means of the Gaussian fits were calculated. Small negative shifts were set to zero, as they are likely to result from variances within noise. Paired recordings and caged-GABA experiments were manually assessed. Network connectivity was estimated by calculating the ratio between connected pairs and the total number of probed pairs. The percentage of action potentials not successfully evoking postsynaptic current responses was determined (synaptic failure rate), as well as the mean amplitude of all successfully evoked postsynaptic responses. Amplitudes of postsynaptic responses were normalized to the first pulse in averaged traces of paired-pulse and short-term plasticity recordings. Individual data points were plotted and linear regression fits were performed using the GraphPad Prism curve fitting toolbox. The mean amplitude of consecutive GABA-uncaging responses was determined in each region (somatic versus dendritic) for each recorded neuron.

Sizes and numbers of immunolabelled gephyrin, GABA_A_Rα2 and GABA_A_Rα5 clusters were assessed using the ImageJ software package (available from http://rsb.info.nih.gov/ij)^6^. In gephyrin/parvalbumin double-staining experiments, parvalbumin-apposed gephyrin clusters were analysed in the stratum pyramidale.

qPCR-data were analysed as described by Pfaffl^71^ with GAPDH serving as reference gene. The qPCR assay efficiency was calculated with the StepOnePlus software (Applied Biosystems) based on a dilution series of five samples for each assay. Western blots were analysed using the Image Studio Software (LICOR).

FRAP of GFP-gephyrin clusters was analysed using the ImageJ software package^17^. Values (corrected for background and bleaching by the imaging procedure) were normalized to prebleach fluorescence and to the first time point after bleaching. All values were expressed as the percentage of the mean prebleach fluorescence (that is, averaged corrected fluorescence of baseline recordings; Δ*t*=5 min) for every analysed cluster (5–12 clusters per culture). FRAP data were fitted using the curve fitting toolbox of GraphPad Prism 6 (GraphPad software, USA) with a bi-exponential equation (two phase association, least squares fit):


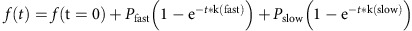


where *P*_fast_ denotes the fraction of the fast component with the recovery time constant *k*_(fast)_ and *P*_slow_ the fraction of the slow component with the recovery time constant *k*_(slow)_, respectively. Owing to difficulties to reliably determine the often rather small *P*_fast_ values observed in some experiments, statistical evaluations were based on comparisons of the averaged FRAP values sampled between 70 and 80 min. Data from non-stimulated pharmacologically treated and untreated cultures were pooled (*c.f.*, Fig. 3e,f and Table 1).

Sample sizes were chosen according to initial pilot experiments and prior studies that used similar experimental approaches. Power estimation was performed using G*Power 3 (Düsseldorf, Germany). Data were analysed using GraphPad Prism 6 (GraphPad software). Statistical comparisons were made using nonparametric tests, since normal distribution could not be assured: Mann–Whitney test (to compare two groups) or the Kruskal–Wallis test followed by Dunn's *post hoc* test, which accounts for multiple testing. *P* values of less than 0.05 were considered a significant difference. All values represent mean±s.e.m. In the figures, * *P*<0.05, **P<0.01 and ***P<0.001; not significant differences are indicated with ‘ns'.

### Digital illustrations

Confocal image stacks were exported as two-dimensional projections and stored as TIFF files. Figures were prepared using the Photoshop graphics software (Adobe, San Jose, CA, USA). Image brightness and contrast were adjusted.

## Additional information

**How to cite this article:** Lenz, M. *et al*. Repetitive magnetic stimulation induces plasticity of inhibitory synapses. *Nat. Commun.* 7:10020 doi: 10.1038/ncomms10020 (2016).

## Supplementary Material

Supplementary Information Supplementary Figures 1-3

## Figures and Tables

**Table 1:**
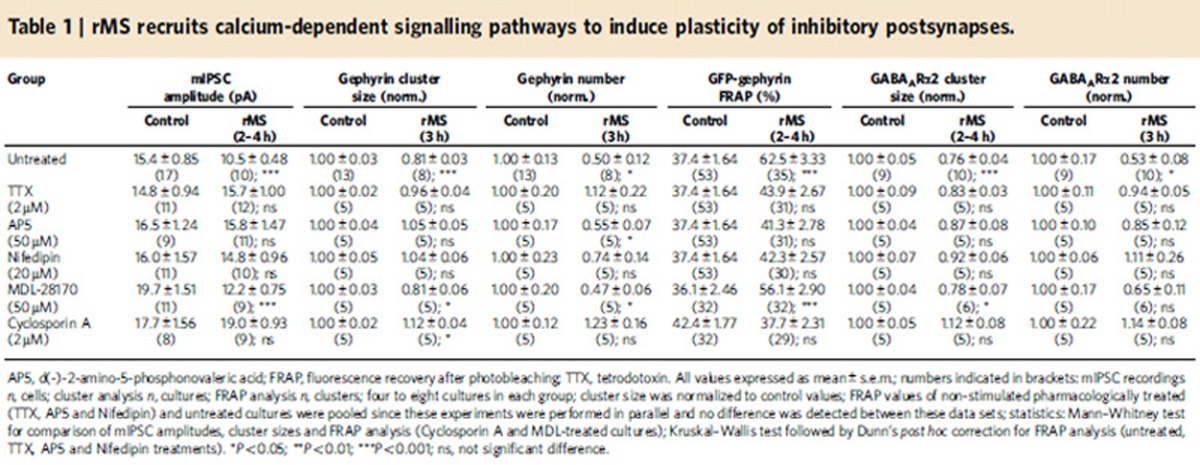
rMS recruits calcium-dependent signalling pathways to induce plasticity of inhibitory postsynapses.
