# Supplementary material for: Repetitive magnetic stimulation induces plasticity of inhibitory synapses
Source: Nat Commun. 2016 Jan 8;7:10020. doi: 10.1038/ncomms10020 (PMC4729863; doi:10.1038/ncomms10020)
Supplement: Supplementary Information — Supplementary Figures 1-3 [file ncomms10020-s1.pdf]

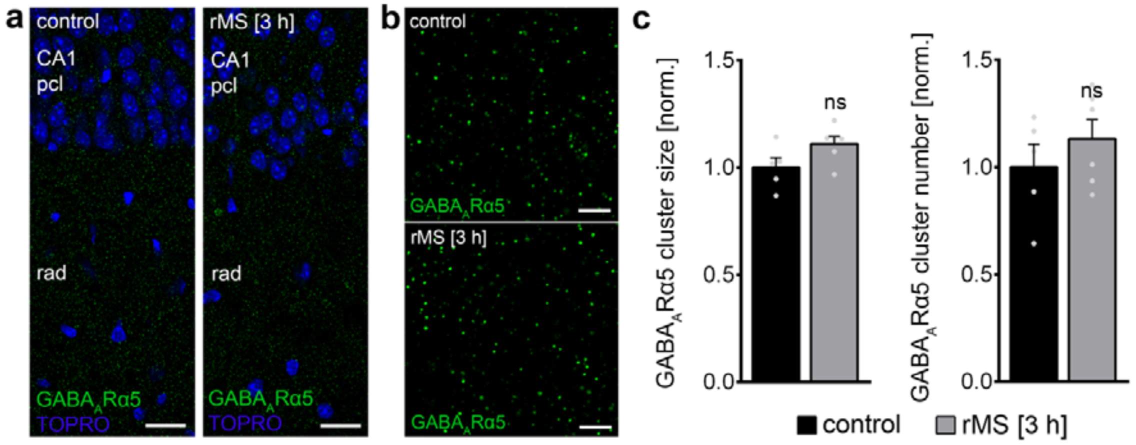

Supplementary Figure 1: rMS does not affect $GABA_A$ receptor subunit α5 clusters

(a, b) Stimulated and non-stimulated slice cultures were stained for $GABA_A$ receptor subunit α5 ($GABA_A$Rα5), which is involved in mediating tonic $GABA_A$R conductance. A clustered $GABA_A$Rα5 pattern was noted in CA1 stratum radiatum (rad) in these experiments, in line with previous work[1,2]. Scale bar in a, 20µm, in b, 4µm.

(c) No significant change in mean $GABA_A$Rα5 cluster size and number was detected at 3h after rMS. Values normalized to non-stimulated control cultures (control, *n*=5 cultures; rMS, *n*=6 cultures; averaged data from three visual fields per culture; Mann-Whitney-test).

Individual data points are indicated by gray dots. Values represent mean±s.e.m. (ns, not significant differences)

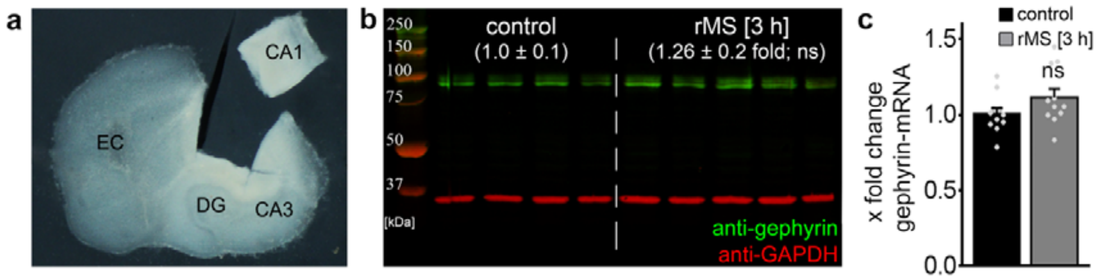

Supplementary Figure 2: Gephyrin expression 3h after rMS

(a) Slice culture tissue containing the CA1 region was isolated and removed with a sterile scalpel from stimulated and non-stimulated slice cultures (3 h after stimulation).

(b, c) The isolated tissue was subjected to Western Blot (b) and qPCR (c) analysis. No significant differences in total gephyrin protein (control, *n*=4 samples; rMS, *n*=5 samples; CA1 regions from two cultures pooled in each sample; Mann-Whitney-test) and gephyrin mRNA levels (*n*=11 cultures per group; Mann-Whitney-test) were observed in these experiments.

Individual data points are indicated by gray dots. Values represent mean±s.e.m. (ns, not significant differences)

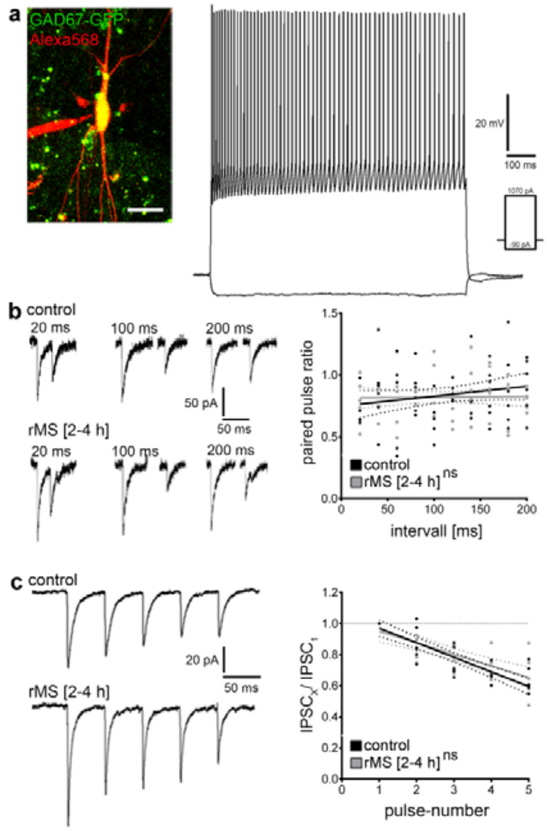

Supplementary Figure 3: Paired recordings in slice cultures of *GAD67-GFP*-mice

(a) Example of a patched and Alexa568 [10µM] filled GFP-expressing interneuron in a slice culture prepared from *GAD67-GFP*-mice. A sample trace illustrating the fast-spiking property of a patched GFP-expressing cell is shown. 1s current square pulses starting at -90pA (ΔI=40pA) were injected in current clamp mode and changes in membrane potential were recorded. Scale bar, 25µm.

(b) Sample traces illustrating paired-pulse responses of CA1 pyramidal neurons at different inter pulse intervals (left). No significant difference between non-stimulated and stimulated cultures in paired-pulse depression is observed 2-4h after stimulation (control, n = 6 cells in 4 cultures; rMS, *n*=5 cells in 3 cultures, linear regression fit, Kruskal-Wallis-test followed by Dunn's post-hoc-test; one data point outside the axis limits).

(c) Sample traces illustrating short-term plasticity tested by 5 consecutive action potentials at a frequency of 20Hz. The amplitude of each consecutively induced inhibitory postsynaptic current was normalized to the amplitude of the first pulse. There was no significant difference between the two groups. (control, *n*=6 cells in 4 cultures; rMS [2-4h], *n*=5 cells in 3 cultures, linear regression fit, Kruskal-Wallis-test followed by Dunn's post-hoc-test).

Individual data points are indicated by gray dots. Values represent mean±s.e.m. (ns, not significant differences)
